# Structure and variability of delay activity in premotor cortex

**DOI:** 10.1371/journal.pcbi.1006808

**Published:** 2019-02-22

**Authors:** Nir Even-Chen, Blue Sheffer, Saurabh Vyas, Stephen I. Ryu, Krishna V. Shenoy

**Affiliations:** 1 Department of Electrical Engineering, Stanford University, Stanford, CA, United States of America; 2 Department of Computer Science, Stanford University, Stanford, CA, United States of America; 3 Department of Bioengineering, Stanford University, Stanford, CA, United States of America; 4 Department of Neurosurgery, Palo Alto Medical Foundation, Palo Alto, CA, United States of America; 5 Department of Neurobiology, Stanford University, Stanford, CA, United States of America; 6 The Bio-X Program, Stanford University, Stanford, CA, United States of America; 7 The Stanford Neurosciences Institute, Stanford University, Stanford, CA, United States of America; 8 Howard Hughes Medical Institute, Stanford University, Stanford, CA, United States of America; Johns Hopkins University, UNITED STATES

## Abstract

Voluntary movements are widely considered to be planned before they are executed. Recent studies have hypothesized that neural activity in motor cortex during preparation acts as an ‘initial condition’ which seeds the proceeding neural dynamics. Here, we studied these initial conditions in detail by investigating 1) the organization of neural states for different reaches and 2) the variance of these neural states from trial to trial. We examined population-level responses in macaque premotor cortex (PMd) during the preparatory stage of an instructed-delay center-out reaching task with dense target configurations. We found that after target onset the neural activity on single trials converges to neural states that have a clear low-dimensional structure which is organized by both the reach endpoint and maximum speed of the following reach. Further, we found that variability of the neural states during preparation resembles the spatial variability of reaches made in the absence of visual feedback: there is less variability in direction than distance in neural state space. We also used offline decoding to understand the implications of this neural population structure for brain-machine interfaces (BMIs). We found that decoding of angle between reaches is dependent on reach distance, while decoding of arc-length is independent. Thus, it might be more appropriate to quantify decoding performance for discrete BMIs by using arc-length between reach end-points rather than the angle between them. Lastly, we show that in contrast to the common notion that direction can better be decoded than distance, their decoding capabilities are comparable. These results provide new insights into the dynamical neural processes that underline motor control and can inform the design of BMIs.

## Author summary

Early studies of premotor cortex explored how individual neurons directly encode aspects of an upcoming movement during preparation. Recent developments have proposed that the dynamics of populations of neurons underlie motor control, and that neural activity during preparation serves to set up these dynamics. While the dynamics of motor control have been studied extensively, several aspects of preparatory activity remain unresolved. Here, we ask how the patterns of neural activity during preparation for different reaches are related to one another. We found that the neural activity during preparation for reaches to different targets has a clear ‘structure’. Additionally, we found that the activity on a given trial was predictive of the initial trajectory of the reach. Lastly, we assessed the implications of our findings for predicting upcoming movements from neural activity, as in brain-machine interfaces.

## Introduction

A central issue in the study of motor preparation has been identifying which aspects of a movement are specified prior to execution and how precisely these aspects are encoded [1–3]. Decades of research have addressed this question with a variety of approaches. Many studies have carefully analyzed aspects of movement execution as a proxy for the preparatory process, for example measuring changes in reaction time to estimate the time it takes to specify different parameters during preparation [1], measuring error patterns in movements made to memorized targets [4–8], or assessing the necessity of preparation in forming motor memories [9]. A potential limitation of using behavioral metrics to measure the preparatory process is the difficulty in delineating which aspects of behavior are related to solely preparation versus movement execution itself [8].

Another prevalent approach for studying motor preparation has been to record neural activity in motor cortex prior to movement execution. Several studies on the neural basis of preparation have used an ‘instructed-delay paradigm’, where a short delay period separates the movement instruction from execution. By determining which aspects of movement are encoded in neural activity during the delay period, the key factors that constitute the preparation process can be inferred. Several studies have shown that the activity of individual neurons in primary motor cortex (M1) and dorsal premotor cortex (PMd) during the delay period correlates with reach direction and distance [10–16], reaction time (the elapsed time between movement instruction and movement onset [17, 18]), maximum speed [16], location of the reach target in visual space [19], target location relative to the eye and hand [20], and many other aspects of the reach. While single-neuron studies have been instrumental in identifying several response properties in delay activity, they also have several limitations (see [21] for review). Most critically, recording neurons one-by-one precludes understanding how neurons covary with one another trial-by-trial, a crucial feature of how neural populations encode information [22].

Over the past two decades, advances in recording technology have enabled the study of populations of simultaneously recorded neurons [23]. Population-level analyses have yielded several advances in the characterization of delay activity, including relating single-trial population activity to reaction time [24], relating responses during the delay period to responses during movement execution [25, 26], and assessing the necessity of the preparatory state [27]. One viewpoint that has emerged from the study of population-level delay activity is the ‘initial condition hypothesis’ of motor preparation, which posits that delay activity sets a preparatory state which seeds movement-related dynamics [18, 24, 28, 29]. While the dynamics of population activity during movement have been studied extensively [26, 29–31], the structure of the neural states that seed these dynamics has not been fully characterized.

In this paper, we seek to understand how the structure and variability of population activity during motor preparation give rise to the upcoming movement. To shed light on this issue, we used multielectrode array recordings from monkeys during an instructed-delay reaching task with a dense target configuration. We first characterized the low-dimensional structure of neural activity in PMd during the delay period by describing how this structure is related to the upcoming reach. Then, we investigated the single-trial variability of that structure and found that it resembles the behavioral variability found in previous studies [6, 7]. Last, we assessed the implication of the neural structure and variability on decoding reach endpoint for brain-machine interfaces.

## Materials and methods

### Ethics statement

All procedures and experiments were approved by the Stanford University Institutional Animal Care and Use Committee. To minimize any potential suffering surgeries were performed under isoflurane anesthesia with carefully monitored post-operative analgesia.

### Experimental design

**Fig 1:**
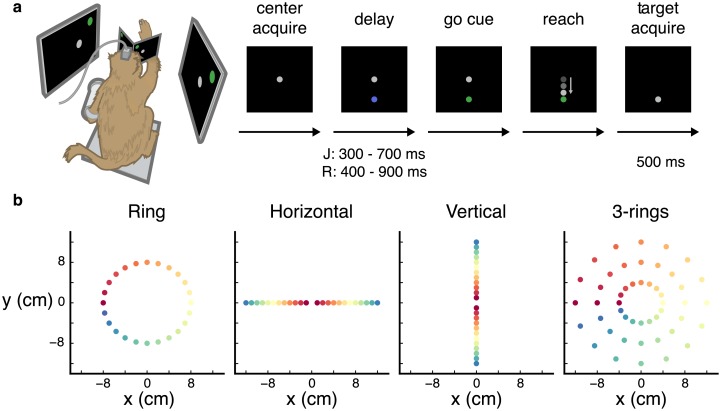
Task timeline and target layouts. (**a**) Experimental setup and timeline of standard instructed-delay reach trial. (**b**) Positions of all targets for four target configurations: ring (24 targets for J; 36 for R), horizontal line (24 targets), vertical line (24 targets) and 3-rings configuration (48 total targets in 3 rings of 16 targets each); only one target is presented to the monkey per trial. Color scheme used for reference in later figures.

In this study, we analyzed neural activity from an instructed-delay reaching task (Fig 1). We trained two monkeys (J and R) to perform center-out-and-back reaches on four different target configurations. In all configurations the target acceptance window was a 2 cm box centered on the target location and the required hold time was 500 ms. Delays were distributed uniformly from 300 to 700 ms for J and 400 to 900 ms for R. To encourge the animals to attend to the stimulus during the minimum delay period, 10% of the trials had no delay. Non-delayed trials were removed from the analysis. Subjects failed the trial if it took longer than 5 seconds to reach the target. This time limit was to stop trials in which the monkey was not attentive to the task. The average reach time of successful trials was J: 582 ms, R: 482 ms and the average reaction time was J: 346 ms, R: 311 ms. Also, the reaction time was negatively correlated (*p* < 1e-4 for both J and R) with the length of the delay period as was shown in previous studies [18]. Each configuration included a high target count to enable accurate quantification of our ability to predict movement endpoint and to have a dense sample of the neural states.

When comparing two different distributions, we used a two-sided Student t-test (assuming unequal variances) with a confidence level of *p* = 0.05 unless otherwise indicated. In figures, we denote * for *p* ≤ 0.05, ** for *p* ≤ 0.01, *** for *p* ≤ 0.001 and **** for *p* ≤ 0.0001; n.s. indicates no significant difference.

All target configurations are presented in Fig 1b. The first target configuration (‘ring’ configuration) varied only in direction. Targets were evenly spaced in a circular formation with a radius of 8 cm; 24 targets for J (6161 trials over four sessions: 2015-10-09, 2015-10-15, 2015-10-26, 2015-10-27) and 36 for R (3577 over two sessions: 2017-01-24, 2017-01-25). The ‘horizontal’ and ‘line’ configurations varied distances and direction; 24 targets were arranged in a line with 12 targets both sides of the center spaced 1 cm apart. For horizontal configuration: 5017 trials over four sessions for J (2015-09-24, 2015-09-25, 2015-09-29, 2015-09-30), 3699 trials over two sessions for R (2017-01-17, 2017-01-18). For vertical: 6110 trials over four sessions (2015-10-01, 2015-10-02, 2015-10-07, 2015-10-08) for J and 4040 trials over two sessions for R (2017-01-15, 2017-01-16). The ‘3-rings’ configuration varied both in direction and in distance, but with more directions and fewer distances (7722 trials over four sessions for J: 2016-01-27, 2016-01-28, 2016-01-29, 2016-02-02) and 912 trials from one session for R: 2017-01-19). Targets were arranged in three concentric rings with radii of 4, 8, and 12 cm, each with 16 targets.

### Neural recording and signal preprocessing

Monkeys J and R were each implanted with two 96-electrodes Utah arrays (Blackrock Microsystems, Inc.), using standard neurosurgical techniques [32] 74 (J) and 64 (R) months prior to this study. The arrays were implanted into the left cortical hemisphere in the dorsal premotor cortex (PMd) and primary motor cortex (M1) as estimated visually from local anatomical landmarks. Monkey R’s array quality is inferior to monkey J’s, and this difference manifests in the results of our study as well as previous studies (e.g. [33, 34]). At the time of the study, monkey R’s M1 array was too poor to use; consequently, in our analyses, we only used the recordings from PMd for both monkeys. We combined recordings from multiple days for all of our analyses. Previous reports have provided evidence for the stability of chronic arrays over days and months [35, 36]. While we do not formally quantify unit stability in the present study, non-stability of the arrays would only add more noise to the data and any unit turnover would end up yielding a null result instead of the results that are presented here. To account for changes in baseline firing rates from day to day, we subtracted from each channel’s instantaneous firing rate its average rate (for that day).

Voltage signals from each of the electrodes were bandpass filtered from 250 to 7500 Hz. Multiunit threshold crossings events were then detected whenever the voltage crossed below a threshold set at the beginning of each day (at −4.5 and −4.0 x Root Mean Square (RMS) voltage for J and R, respectively). Contralateral hand position was measured with an infrared reflective bead tracking system (Polaris, Northern Digital) polling at 60 frames/s. All analyses were performed offline after data collection. Threshold crossings recorded on the array’s 96 electrodes (i.e., channels) were binned during the 200 ms prior to the go cue. To characterize the quality of each channel, we fit a regression from each channel separately to the task (x, y location of the target). Of the 96 electrodes, we found that 74 for J and 80 for R had significant correlation (*p* < .05, with Bonferroni correction where n = 96) with the task.

### Dimensionality reduction

In this work, we used dimensionality reduction to analyze patterns across the neural population that are not apparent at the level of individual neurons. Our overall aim is to identify prominent structure in population activity and see how this structure relates to the task and movement.

Our data begins as an order-3 tensor $N\in R^{n\times t\times k}$ where *n* is the number of recorded units, *t* is the number of timesteps recorded, and *k* is the total number of trials across all tasks and experimental sessions. We first binned the data by taking the average firing rate in a 200 ms time window before the go cue (during the delay period), leaving a matrix $N_{delay}\in R^{n\times k}$. To focus our investigation on the dimensions that capture the majority of the variance in our data, we used principal components analysis (PCA), an unsupervised technique. In order to find dimensions that capture variability related to differences between conditions rather than within-condition variability, we performed PCA using the condition-averaged data. That is, we performed PCA using $N_{\text{condition-average}}\in R^{n\times c}$, where *c* is the number of distinct reach conditions (i.e. targets) across all tasks (J: 120, R: 132), mean-centering across conditions before applying PCA. This yields a linear transformation $A\in R^{d\times n}$ where *d* is the number of reduced dimensions. The top 6 PCs explained the following cumulative percentages of the variance: 58% 82% 88% 92% 94% 95% (J); 81% 89% 92% 94% 95% 96% (R). We kept the top 6, 5 (J, R) principal components in order to retain 95% percent of the variance. Though we performed PCA using the condition-averaged data, we used the resulting *A* transformation to reduce the dimensionality of individual trials (i.e. *AN*_*delay*_).

After reducing dimensionality with PCA, we rotated and scaled (using regression) the principal components to find the subspace that best explains the *x* and *y* position of the target. Concretely, we found *β*_*x*,*y*_

$$\begin{array}{c} {}[\begin{array}{c} x_{neural}\\ y_{neural} \end{array}]=\beta_{x,y}AN_{delay} \end{array}$$ (1)

where *β*_*x*,*y*_ minimizes the squared error between $\left[\begin{array}{c} x_{neural}\\ y_{neural} \end{array}\right]$ and the target location $\left[\begin{array}{c} x\\ y \end{array}\right]$. In order to compare potential coordinate systems, we also regressed to direction (represented as a unit vector in the target direction) and distance separately.

Additionally, we found a projection that best explains the maximum speed achieved during each reach. Unlike in the regression to target location, for the speed projection, we regressed directly from the neural activity rather than the reduced-dimensional data. Since the PCs were derived using condition-averages of the neural data, trial-to-trial variability related to speed was averaged over; repeating PCA on condition-averages for speed would have required explicit conditions for different speeds. Thus, the equations defining the speed regression:

$$\begin{array}{c} {}[\begin{array}{c} speed_{neural} \end{array}]=\beta_{speed}N_{delay} \end{array}$$ (2)

where *β*_*speed*_ is chosen to minimize squared error between [*speed_neural_*] and the true maximum speed on each trial.

In order to maintain independent dimensions (as in PCA), we orthogonalized the coefficients of the linear mapping for each variable (*x*, *y* and speed). This was done by sequentially fitting the regression coefficients (for *x*, then *y*, then speed), but between each fit, we projected the neural data into the null-space of the previous regression coefficients. In practice, this process did not change the projections much, as the projections found without orthogonalization were also nearly orthogonal.

### Classification with support vector machines (SVMs)

For classification, we used support vector machines (SVMs). SVMs are classifiers that construct a separating hyperplane between data points belonging to different classes, making a decision boundary with the greatest distance between the boundary and the data points. Multiclass classification was handled with one-vs-one classification. We used the scikit learn library implementations of SVMs [37]. We found the best kernel for the SVM to be a radial-basis function. All hyperparameter searches were performed on a held-out dataset of 2237 trials from the 3-rings task configuration. All reported classification accuracies are the mean accuracy across 10-fold cross validation, and errors are the standard error of the mean (SEM) across folds.

## Results

### Structure of neural activity during delay period

**Fig 2:**
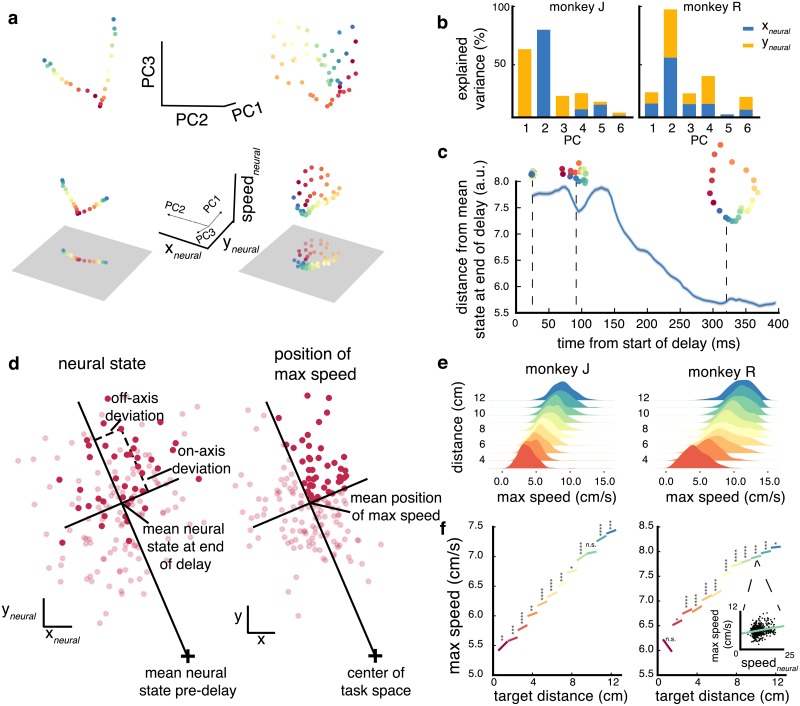
Structure of delay activity and relation to behavior. (**a**) Projection of condition-averaged delay activity onto top 3 PCs (top row) and *x*_*neural*_, *y*_*neural*_ and *speed*_*neural*_ (bottom row) dimensions for the horizontal line (left column) and 3-rings (right column) target configurations for monkey J. Bottom gray square shows 2D projection onto the *x*_*neural*_, *y*_*neural*_ plane. The top 3 PCs are shown in relation to the spatial plane in the axes of the bottom row. (**b**) Explained variance of *x*_*neural*_ and *y*_*neural*_ dimensions by each of first 6 principal components. (**c**) Time-course of the neural state in the spatial plane (monkey J). Blue line shows average distance of the neural state from the converged delay state. Projections onto the spatial plane throughout the time-course are shown above. (**d**) Single-trial neural state correlates with initial reach kinematics. Right shows *x*, *y* position of maximum speed for reaches to one target in the 3-rings configuration (monkey J). Trials where position of maximum speed falls within top-right quadrant of on/off axes are opaque. Left shows corresponding neural states for those reaches with the same trials opaque as on right; these neural states tend to fall within same quadrant. (**e**) Each density shows the distribution of maximum speeds for reaches to a given distance in vertical configuration; reach distance correlates with max speed but there is variability of speed within each distance. (**f**) *speed*_*neural*_ component of neural state is correlated with max speed for reaches to same distance (data combined across horizontal and vertical configurations). Regression was fit for each reach distance separately, confirming that this axis encodes speed independent of target distance.

To study the structure of delay activity in PMd across different reaching conditions, we represented population responses during the delay period of a trial (the average activity in the last 200 ms) as a point in the 96-dimensional neural state space. We used PCA on the combined datasets (tasks and sessions) to identify primary patterns of activity in an unsupervised fashion. Projecting the condition-averaged neural activity to the top principal components revealed a clear 3D structure between neural states for different reaches (top 3 PCs for monkey J shown in Fig 2a, top row). In one plane the condition-average neural states appeared to be organized by their target position, and in an orthogonal dimension by the distance of the upcoming reach. These apparent dimensions were mixed among the top principal components. In order to reason about this structure quantitatively, we would like to define axes that are aligned with these dimensions. To do so, we simply rotated the space defined by the top PCs using regression to task variables (see Methods). However, it is not entirely clear which aspects of movement the neural states are organized by, and thus which variables to regress to. In particular, we cannot infer from the condition averages alone whether the structure is related to task/visual parameters (e.g. location or distance of the target) or a plan for the upcoming movement (reach endpoint, speed). To disambiguate between these possibilities, we 1) compared different candidate coordinate systems and 2) show that the single-trial neural state correlates with aspects of the reach, suggesting that the structure is related to the plan for the upcoming movement.

We first oriented the space with respect to the *x*, *y* position of the target by defining a ‘spatial plane’. To define the ‘spatial plane’ (Fig 2a bottom row, gray plane) which reflects the *x*, *y* position of the target, we used linear regression on the top PCs (6 for J, 5 for R; see Methods) to yield two orthogonal axes, *x*_*neural*_, and *y*_*neural*_. Thus, *x*_*neural*_, and *y*_*neural*_ are each just a linear combination of the top PCs (Fig 2b). The spatial plane captures a large percentage of the overall variance of the trial-averaged neural responses for the ring, horizontal, vertical, and 3-rings tasks: 66.8%, 64.4%, 47.7%, and 68.2% (monkey J), 33.7%, 30.7%, 31.0%, 30.1% (monkey R) for each task, respectively (*p* < 1e-4 for both monkeys). The difference between the monkeys is likely a result of the array quality difference (see Methods). Interestingly, at target onset, the neural states projected onto this plane start at the center of this plane, start to diverge after 50 ms (R: 75 ms), and converge to the steady neural states after J: 300 ms, R: 400ms (Fig 2c). It is worth noting that reach direction and distance are encoded together as *x*, *y* coordinates, rather than in orthogonal subspaces. This joint encoding was not simply a consequence of the method of constructing this subspace (regressing to *x*, *y*); even when the population activity is regressed only to a unit vector in the target direction (without distance information), the resulting neural states are structured nearly identically to the structure in Fig 2a.

To show that the neural state on the spatial plane is predictive of the upcoming reach and is not merely a result of the visual stimuli, we examined the relationship between the neural states and reaches on individual trials. In particular, we performed a trial-by-trial correlation between on-/off-axis variability of the neural state and on-/off-axis kinematic variability. Rather than measure kinematic variability at the endpoint of the reach (which is influenced by visual feedback), we measured variation at the early, largely feedforward, part of the reach. Previous work demonstrated that the spatial position of maximum speed is predictive of reach endpoint in the absence of visual feedback [8], so we compared the deviations in the neural state space to deviations in the spatial position of maximum speed (Fig 2d). By deviations, we mean the displacement of a trial from their condition average: the average neural state or the average position of maximum speed for reaches to a given target. To disambiguate deviations in distance from deviations in direction, we decomposed the deviations into two components: ‘on-axis’ deviations, which measures variation in distance, and ‘off-axis’ deviations, which measure variation in distance and direction, respectively [6, 7]. Both on-axis (*p* = 1e-3 for J, 4e-3 for R) and off-axis (*p* = 4e-4 for J, 2e-4 for R) deviations in the neural state were positively correlated with on-axis and off-axis deviations in the position of max-speed for both monkeys. Thus, the neural state in the spatial plane is predictive of the maximum-speed position (and the planned end-point position) of the upcoming reach.

Next, we investigated the third dimension, on which the neural states are organized based on the (absolute) target distance. During natural reaches, reach distance and maximum speed are known to be highly correlated [6, 38]; thus, it is unclear from the structure seen in the condition-averaged neural states whether this third dimension encodes target distance or reach speed. We resolved this ambiguity by testing whether the neural state on single trials carries speed-specific information. For reaches to a given target distance, there is trial-to-trial variability of maximum speed. If speed is encoded in delay activity, the neural state on individual trials should covary with speed even for reaches to the same target distance. Thus, we controlled for the correlation with distance by examining whether the neural state on single trials carries information related to speed. We first defined a 1-dimensional projection of the neural activity (similar to the construction of the spatial plane) by regressing between the high-dimensional neural state and the single-trial maximum speed. We then tested whether the neural state in this dimension varied with speed for reaches to the same target, and found that for almost all distances it did (J: 11/12 distances in the vertical and horizontal tasks, R: 11/12, *p* < 0.05), confirming that this axis encodes speed independently of target distance (Fig 2f). However, this does not preclude the existence of (speed-independent) distance related information; indeed, we found that after regressing out speed from the neural activity, there is still a positive correlation (*p* < 1e-4 for J and R) with target distance. While both speed and distance dimensions exist in the neural activity, the structure observed in the top principal components (Fig 2a) more strongly reflects the speed dimension. That is, when projecting the speed and distance dimensions onto the top PCs, the distance projection is approximately half compared to the projection of the speed dimension. We note that this test was possible without instructing slow/fast reach conditions as in [16] because the simultaneous recording of many neurons afforded us the statistical power to estimate speed from neural activity. However, due to the lack of reach-speed conditions, we cannot report the percentage of variance this dimension captures for condition-averaged neural states (as we did for the spatial plane). This dimension explains J: 2%, R: 1% of the variance in the neural state across all trials; for reference, the spatial plane explains J: 20%, R: 10% for single trials, lower than the condition averages due to within-condition variability.

### Neural state variability during delay

Previous behavioral studies have provided detailed quantification of the variability of motor preparation by using reaches made in the absence of visual feedback as a proxy for the prepared movement [6, 7]. Their main findings were that the variability ‘on-axis’ (distance) was larger than ‘off-axis’ (direction), and that these variabilities decreased (relative to reach distance) as reach distance increased. In the present study, we can observe the trial-by-trial neural state during preparation directly, and measure variance within the spatial plane described in the previous section. In the following section, we test whether the properties of preparation variability observed behaviorally are also observed in delay activity by performing analyses parallel to the referenced behavioral studies but in neural state space.

**Fig 3:**
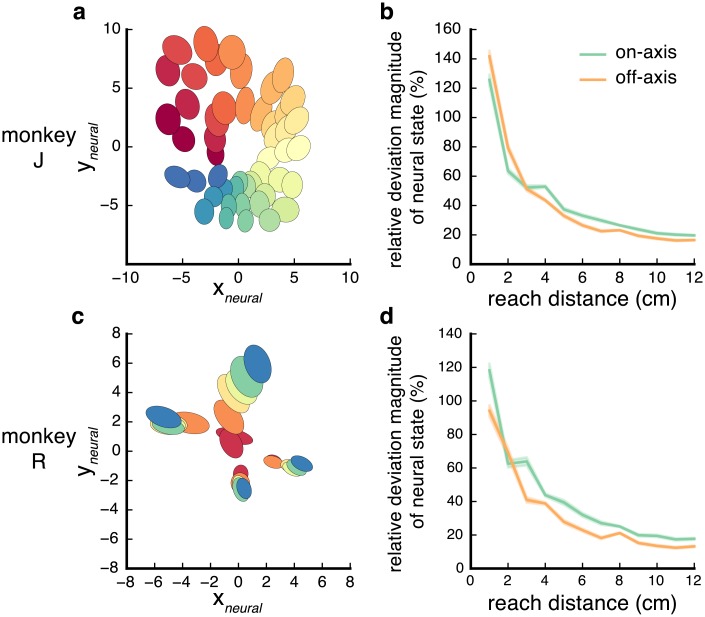
Variance of neural state in spatial dimensions. (**a**) Equal frequency ellipses for the distributions of neural states for each target in the 3-ring task (monkey J). The major axis of each ellipses is the direction of maximal variance in the distribution. The ellipses axes were scaled to capture 95% of the data then scaled down by a factor of 6 for visual clarity; the orientation and relative lengths of the axes are preserved. (**b**) Magnitude (absolute value) of on-axis (green) and off-axis (orange) deviations as a function of reach distance; data taken from all tasks (monkey J). Vertical axis is expressed as a percentage of reach distance. Thick line shows average across all trials of the same distance, shaded boundary shows ± SEM across trials. (**c, d**) Same as panels **a** and **b** but for monkey R.

We characterized ‘spatial’ distributions in neural state space in a similar manner to how [6] and [7] measure endpoint distributions. First, we used PCA to determine the axis of maximal variance for each reach condition. We visualized the distribution for each condition as an ellipse whose major and minor axes correspond to the first and second principal components, respectively; distributions for the 3-ring task are shown in Fig 3a. The axes of the ellipses are scaled relative such that, on average, 95% of the neural states fall within the ellipse. Much like the endpoint distributions of reaches made in the absence of visual feedback in previous behavioral studies [6–8], we found that the ellipses for most conditions were oriented in the direction of the mean neural state from the origin. To quantify the difference in distance and direction variability, we decomposed the deviations of single trials into their ‘on-axis’ (distance) and ‘off-axis’ (direction) components (as in the previous section) and calculated the overall variance of these components separately. The mean absolute on-axis deviation for each target and for each monkey was systematically greater than the mean absolute off-axis deviations in the tasks (*p* = 9e-7 (ring), 4e-7 (horizontal), 1e-8 (vertical), and 3e-14 (3-rings) for J, 8e-3, 4e-7, 2e-7, 0.19 for R). We did not observe a significant result in monkey R 3-ring task likely due to the significantly smaller trial count. In sum, deviations in distance were larger than deviations in direction.

In addition to showing that the spatial distributions of reach endpoints are elliptical, [6] and [7] also analyzed the influence of movement distance on variability in both direction and distance. In both studies, they computed the *relative* error, meaning the deviation normalized by reach distance. They found that the relative errors in both direction and distance decreased as reach distance increased. To assess whether the neural state variability is likewise influenced by reach distance, we performed similar analyses by testing whether on-axis and off-axis deviations in the neural state were affected by reach distance. Similar to those studies, we found that the relative deviations in both distance and direction in the neural state decreased as a function of reach distance (Fig 3b and 3d). Thus, several properties of the variance of reach endpoint [6, 7] are likewise observed in the neural state preceding reach.

### Accuracy of endpoint prediction from delay activity

In the previous sections, we looked at the structure and variance of delay activity via dimensionality reduction. We next sought to understand how this structure and variance affects the accuracy of decoding cued target location from delay activity, as in discrete brain-machine interfaces (BMIs) [39–41]). Most studies describe distance and direction as the main two factors that are prepared for the upcoming reach. Previous single-neuron studies have suggested that neurons have weaker tuning for distance compared to direction [14, 16]. Following this observation, BMI studies have typically used radial target layouts to maximize correct predictions (and communication rate, e.g. [40]). However, our results on the structure and variance of delay activity imply that measuring decoding accuracy for direction and distance separately may be misguided; for example, from Fig 3a it seems that distinguishing between two targets with the same angular separation would be easier at further distances.

**Fig 4:**
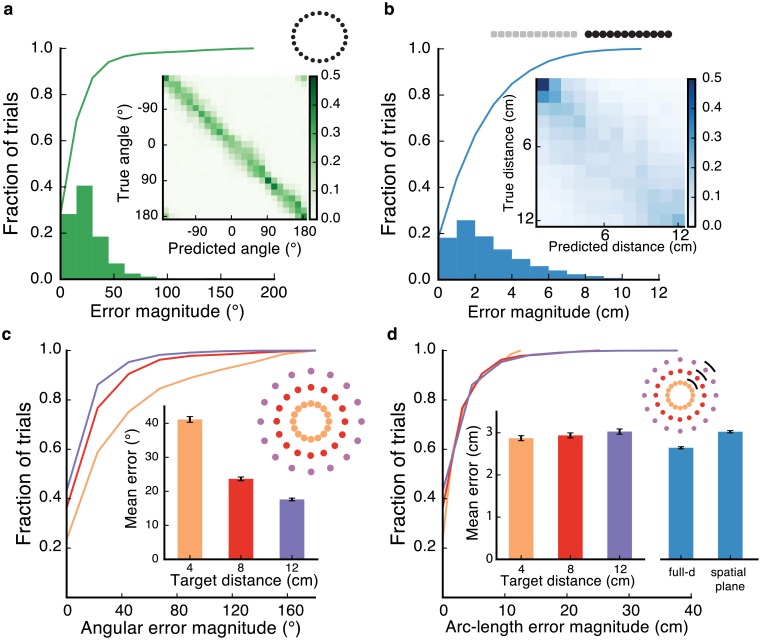
Quantifying endpoint information content in motor preparatory activity, Monkey J. (**a**) Direction classification (8 cm ring of 24 targets ‘ring’ task). Histogram of residual angles (absolute angle difference between true and predicted direction). Line shows cumulative total fraction of classified trials. Inset shows confusion matrix for classification. (**b**) Distance classification. Data combined from both horizontal and vertical target configurations. Both target arrangements have 12 targets on two sides of center (up/down or left/right), but the classifier was trained and tested on only one direction at a time; the results are aggregated across directions. Results are shown as in **a**. (**c**) Cumulative distribution of angle errors for direction classification (16 targets) at 3 distances. Inset shows mean angle error. (**d**) Same as **c**, but error is reported in arc-length instead of angle. Arcs of equal length are displayed in black for each of the 3 rings. Inset shows mean arc error; for the 8 and 12 cm rings, this mean was computed using only errors within the range of possible errors for the 4 cm ring. Left blue bar shows mean error in distance classification (i.e. the mean of the errors in **b**); right blue bar shows mean error in distance classification when neural data is projected to spatial plane before classification.

If we compare decoding accuracy for direction v.s. distance, the results can be hard to interpret or misleading. For example, when we compared the classification accuracies for classifying the ring and horizontal/vertical datasets (using SVMs—see Methods), direction was J:32.2% ± 0.6% accuracy for 24 targets (R:16% ±.5% for 36 targets) while distance classification for only 12 targets was lower for J (18.9% ±.1%) and comparable for R (21.4% ±.2% for R). As classification accuracy depends on the number of targets, we can instead compare the distribution of classification errors, which gives a more complete description of the classifier’s performance than overall accuracy and is less sensitive to the total number of targets. In comparing the error distributions (Fig 4a and 4b), distance classification appears to suffer from more large-magnitude errors than direction. However, from Fig 3 it seems that distinguishing between two targets with the same angular separation would be easier at further distances. Indeed, classifying direction yields higher classification accuracy at further distances (Fig 4c); when classifying targets in 3-rings separately for each distance, accuracy increases with ring distance (23%, 36%, 43% for J; 22%, 28%, 29% for R). Thus, quantifying decoding performance in terms of direction and distance is misleading, as classification accuracy for direction decoding *depends* on reach distance.

Two factors without interaction would be a better choice from the point of view of quantifying decoder performance, and could be useful for designing decoders or target interfaces. Surprisingly, we found that quantifying directional decoding performance in terms of arc-length error provides a measure that is independent of target distance. When presenting the decoding accuracy in term of angular error, mean error decreases as a function target distance (Fig 4c). In contrast, when presenting the errors as arc-length (scaling the angle based on the radius), the arc-length error distributions are not significantly different (F test; *p* > .05 for both J and R), i.e. the arc-length errors are distance independent. Thus, arc-length provides a measure of decoder performance that is independent of distance.

Another benefit of presenting the directional decoding accuracy as arc-length is that it has the same unit as distance (i.e. cm) permitting direct comparison between the two. We plotted the mean error from the distance classification in Fig 4d. Surprisingly, we found that the mean classification error for distance classification was significantly (albeit only slightly) *lower* than the errors for directional classification. In contrast to previous claims [40], this result suggests that decoding distance from delay activity is at least as accurate as decoding direction. This is surprising given the difference in variance between distance and direction in Fig 3, but may be explained by the presence of information related to speed during preparation [16] which is correlated with distance during natural reaches [6, 38]. To test this, we projected the delay activity to the spatial plane (shown in Fig 2) prior to classification to remove speed information. The resulting classification error rate (Fig 4d, blue bars) is significantly greater than classification using the full dimensional neural activity (3.03 cm v.s. 2.64 cm J, 2.78 cm v.s. 3.27 cm R; *p* < .05 for J and R), and is not significantly different than the directional classification errors (*p* >. 05 for each distance in J and R). This finding is odd given the structure of the variances of the previous section. The SVM algorithm is likely unable to take advantage of the albeit modest differences between the variances. Perhaps future work that employs non-linear methods that specifically take advantage of this difference might show more changes.

## Discussion

### Does delay activity represent kinematic variables?

Reaching to a visual target necessarily involves a series of visuomotor transformations beginning in the coordinate system of the retina and resulting in an appropriate motor response. The details of when and where visual information is transformed into abstract kinematics, action selection, and movement specification have yet to be settled. Several observations have been consistent with PMd encoding ‘high-level’ aspects of reaching not directly related to movement specification: neurons in PMd respond selectively for the location of the target in visual space [19], correlate with upcoming reaches with the ipsilateral arm [42], and represent movement in a variety of reference frames [20]. Other studies have shown that PMd activity during movement changes when reach kinematics are fixed but muscle activity differs, suggesting that neurons in PMd support ‘low-level’ movement specification [43].

While we describe population activity with reference to kinematic variables, it is not the aim of our study to provide evidence for whether PMd encodes abstract kinematics or muscle-like commands. Instead, the primary goal of this work is to determine the structure of population-level delay activity; we use the language of abstract kinematic variables (*x*, *y* position of reach endpoint and maximum speed) to describe the subspace of delay activity, but we note that these abstract variables are correlated with lower-level aspects of movement [44, 45]. To better assess whether the structure observed in delay activity more strongly resembles abstract kinematics or muscle commands, future work could employ a similar strategy to [43] whereby subjects make reaches with similar kinematics (e.g. hand paths) but with different muscle forces (e.g. arm postures). If the population responses retain the structure in Fig 2a despite different muscle output, this would support the notion that delay activity encodes abstract kinematic variables. If, instead, the organization changes or delay activity occupies a different subspace when movement specification changes, then delay activity is likely more related to the particular muscle activations or joint trajectories required for movement execution.

### Independence of direction and distance

The issue of whether direction and distance are ‘independent’ quantities during motor planning has long been a subject of debate in both behavioral and neurophysiological studies. Our study provides further details about the relationship between direction and distance by examining their representations at the population level. First, as is discussed in the results, the neural states for reaches to different targets are organized according to reach endpoint (in Cartesian coordinates), rather than distance and direction separately. Indeed, when we searched for a subspace related to solely direction (without distance information), we could not find such a subspace, suggesting that direction is only encoded in conjunction with distance. However, it is clear from the distribution of neural states within these dimensions (Fig 3) that direction and distance have different noise properties—The encoding of distance in the neural state is more variable than the encoding for direction. Thus, there is still a notion of direction and distance as separate quantities in the population. In sum, our results suggest that the relationship between direction and distance encoding in planning activity is not simply ‘independence’ or ‘dependence’, but a combination of the two.

### Organization of initial conditions

The ‘initial condition hypothesis’ of motor preparation posits that the preparatory state seeds movement-related dynamics [18, 24, 28, 29]. One way to view the present work is as an extension of the initial condition hypothesis: we describe how the initial conditions for different reaches are organized with respect to one another. The radial organization of neural states had been previously reported in studies using center-out reaches that varied solely in direction [26, 34, 46]. By using target layouts that varied in both direction and distance, we observed further organization of the neural states by 1) the *x*, *y* location of the reach endpoint and 2) maximum speed. Though the organization of these initial conditions provides a signature of the dynamical system, much work remains before the dynamics of movement planning are fully characterized. In particular, while we have described a few primary components of delay activity, a comprehensive model needs to fully describe the relationship between other aspects of movement (e.g. reaction time [24], posture [47]) and the structure described here. Additionally, we hope that future studies will investigate the computational purpose of the organization of delay activity. For example, future computational experiments could assess under which task conditions this structure is observed by e.g. training a recurrent neural network to perform similar tasks [26, 31, 48].

### How does the neural space scale with task complexity?

In our work we sought to understand a simple 2D center-out reach task. We found that the main three parameters encoded in PMd during delay activity are *x*, *y* position of reach endpoint and maximum speed, which are a sufficient set of parameters to specify a ballistic reach. The low-dimensionality of the neural space might be a result of the low dimensionality of the task. We believe that the characterization of how neural states are distributed within these dimensions is of value, both for insights into how behavioral variability is generated and for improving decoding for BMIs. In more complex tasks such as reaching in high dimensional space, reaching with obstacles, movement in a maze, or a sequence of reaches, a larger set of parameters are required to define the upcoming reach (such as the curvature or additional dimensions for the spatial subspace). In those tasks, we might expect to encounter neural states during the delay period with a greater number of dimensions than observed in the present study [49], and we expect that using similar approaches to those employed in our study with higher dimensional tasks will lead to further insights.

### Delay activity and motor preparation

We, and many others, have observed correlations between neural activity during the delay period and aspects of the subsequent reach. In particular, we have shown that neural states during the delay are predictive of the upcoming movement on single trials and that ‘spatial’ variability of these states is similar to the variability observed in behavioral studies in the absence of visual feedback [6–8]. The resemblance between the variability of the neural state and the variability of movement suggests that the movement variability is a consequence of the preparatory process rather than e.g. properties of movement execution. That said, our results do not provide a causal link between the delay state and the following movement; indeed, recent studies have questioned the necessity of delay activity for motor preparation [27]. To concretely demonstrate that movement execution is determined by neural activity during the delay, future studies could perturb delay activity along specific dimensions in the neural state (Fig 2a) and observe whether the following reach is similarly perturbed.

### Applications to brain-machine interfaces

Our results show that direction and distance are decodable with comparable accuracy during movement preparation, suggesting that discrete BMI performance is likely affected by the total spatial distance between targets. Discrete decoders, which use neural activity to classify the most likely movement endpoint from a discrete set of possible endpoints, show promising results for communication [39–41, 50]. We emphasize that just because decoding accuracy depends on the total spatial distance between targets does not imply that activity in PMd solely encodes reach endpoint. Instead, we propose that the encoding of distance is more variable than direction (Fig 3), but the speed dimension (Fig 2a) provides additional information which makes up for this disparity. We confirm this by demonstrating that when speed information is removed, distance decoding worsens (Fig 4d). It follows that this should only be the case because of the correlation between reach distance and speed during natural reaches. If future work did similar experiments but with different instructed speeds (e.g. requiring slow movement to further targets and quick movement to nearby targets, thus reversing the typical correlation between speed and distance), we would predict that direction would indeed be more decodable than distance. We also acknowledge that prior studies [51] have compared direction and speed decoding from neural activity during movement execution and concluded that direction is decodable with higher accuracy than speed. Thus, our finding that direction and distance are decodable with comparable accuracy may be specific to delay activity, and thus may be more applicable for discrete BMIs. Nonetheless, it would be interesting for future studies to attempt to better understand why these differences exists between movement preparation and execution.

In prior work on target-layout optimization for BMIs [52], gains in decoder performance were proposed by optimizing target placement based on the tuning properties scaled by distance of the recorded neurons. Based on our results, a model that also includes a separate distance term (which can be indirectly influenced from planned max velocity), in addition to the *x*, *y* location of the target, might increase decoding performance and better utilize a given workspace for communication. Future experiments with different target layouts could verify the optimal target layout.
